# Reciprocity between Regulatory T Cells and Th17 Cells: Relevance to Polarized Immunity in Leprosy

**DOI:** 10.1371/journal.pntd.0004338

**Published:** 2016-01-11

**Authors:** Soumi Sadhu, Binod Kumar Khaitan, Beenu Joshi, Utpal Sengupta, Arvind Kumar Nautiyal, Dipendra Kumar Mitra

**Affiliations:** 1 Department of Transplant Immunology and Immunogenetics, AIIMS, New Delhi, India; 2 Department of Dermatology and Venereology, AIIMS, New Delhi, India; 3 Immunology Division, National Jalma Institute for Leprosy and Other Mycobacterial Diseases, ICMR, Agra, India; 4 Stanley Browne Research Laboratory, The Leprosy Mission, Shahdara, New Delhi, India; Fondation Raoul Follereau, FRANCE

## Abstract

T cell defect is a common feature in lepromatous or borderline lepromatous leprosy (LL/BL) patients in contrast to tuberculoid or borderline tuberculoid type (TT/BT) patients. Tuberculoid leprosy is characterized by strong Th1-type cell response with localized lesions whereas lepromatous leprosy is hallmarked by its selective *Mycobacterium leprae* specific T cell anergy leading to disseminated and progressive disease. FoxP3+ Regulatory T cells (Treg) which are essential for maintaining peripheral tolerance, preventing autoimmune diseases and limiting chronic inflammatory diseases also dampen proinflammatory T cells that include T helper 17 (Th17) cells. This study is aimed at evaluating the role of Treg cells in influencing other effector T cells and its relationship with the cytokine polarized state in leprosy patients. Peripheral blood mononuclear cells from of BT/TT (n = 15) and BL/LL (n = 15) patients were stimulated with *M*. *leprae* antigen (WCL) in presence of golgi transport inhibitor monensin for FACS based intracellular cytokine estimation. The frequency of Treg cells showed >5-fold increase in BL/LL in comparison to BT/TT and healthy contacts. These cells produced suppressive cytokine, IL-10 in BL/LL as opposed to BT/TT (*p = 0*.*0200*) indicating their suppressive function. The frequency of Th17 cells (CD4, CD45RO, IL-17) was, however, higher in BT/TT. Significant negative correlation (r = -0.68, P = 0.03) was also found between IL-10 of Treg cells and IL-17+ T cells in BL/LL. Blocking IL-10/TGF-β restored the IL-17+ T cells in BL/LL patients. Simultaneously, presence of Th17 related cytokines (TGF-β, IL-6, IL-17 and IL-23) decreased the number of FoxP3+ Treg cells concomitantly increasing IL-17 producing CD4+ cells in lepromatous leprosy. Higher frequency of Programmed Death-1/PD-1+ Treg cells and its ligand, PDL-1 in antigen presenting cells (APCs) was found in BL/LL patients. Inhibition of this pathway led to rescue of IFN-γ and IL-17 producing T cells. Results indicate that Treg cells are largely responsible for the kind of immunosuppression observed in BL/LL patients. This study also proves that Treg cells are profoundly affected by the cytokine milieu and this property may be utilized for benefit of the host.

## Author Summary

Polarized T cell response (Th1/Th2 biased) to *Mycobacterium leprae (M*. *leprae)* is believed to be a critical element in the pathogenesis of leprosy and its varied clinical manifestations. However, immune response at the pathologic sites of leprosy is an extremely complex process, particularly in the light of recently evidenced heterogeneity of T cell subsets. FoxP3 positive regulatory T cells (Treg) are one of the most potent hierarchic cell types suppressing the effector T cell function with eventual regulation of immune response elicited by the host during intracellular infections. This study shows the recovery of the cell mediated response by CD4+ T cells by inhibiting the suppressive cytokines, IL-10 and TGF-β and also by blocking of the Programmed Death-1 pathway in cells isolated from lepromatous leprosy patients. Reversal of IL-17 immune response was also achieved by modulating the cytokine milieu of *in vitro* cell culture and hence provides us cues to counter the *M*. *leprae* unresponsiveness in leprosy patients.

## Introduction

Leprosy is a disease of immunological spectrum tightly correlating with the extent of pathology and clinical manifestation [1]. It is well known that T cell defect is a distinctive feature in lepromatous leprosy (LL) in contrast to that of tuberculoid leprosy (TT) patients. In between these clinical entities lie borderline tuberculoid (BT), borderline lepromatous (BL) and borderline borderline (BB) all displaying symptoms in between the two polarized forms [2]. Selective T cell unresponsiveness to the antigens of *M*. *leprae* occurs among LL patients, while responsiveness to several other antigens remains intact, a phenomenon known as “split anergy” [3]. BT/TT patients with strong T cell reactivity against *M*. *leprae* is associated with biased production of IFN-γ dominant immune response, while BL/LL patients, so called anergic and disseminated form of the disease demonstrates T cell response skewed towards IL-4 and/or IL-10 dominant cytokine production [4]. Polarized immunity against *M*. *leprae* is a critical element in the pathogenesis of leprosy and plays an important role in the varied clinical manifestations of leprosy [5]. Biased cytokine production has also been documented at the lesional levels of both TT as well as LL forms of leprosy [6]. However, generation of Th1/Th2-like effector cells alone cannot fully explain the polarized state of immunity. Other subsets of T cells have been identified which play important role in determining host immunity [7,8].

Lately, FoxP3 positive regulatory T cells (Tregs) have been characterized as one of the most potent hierarchic cell type suppressing effector T cell function with eventual regulation of immune response elicited by the host during intracellular infections such as tuberculosis [9] and leishmaniasis [10,11]. The CD4+CD25+ natural regulatory Treg cells expressing the transcription factor forkhead box P3 (FoxP3) is the best characterized suppressive T-cell subset [12]. These cells are critical for the maintenance of self-tolerance and play an important role in a wide range of clinical conditions such as autoimmune diseases, transplantation rejection reactions, cancer, as well as infectious diseases [13,14]. Mediators of Treg-cell induced suppression include the inhibitory cytokines, IL‑10 and TGF-β [15,16]. Over representation of Treg cells in the periphery and particularly at the pathologic sites of infection has been shown to be critical in determining local immunity, thus dictating the outcome of the disease among patients suffering from various forms of tuberculosis [9]. Recently, it was revealed that FoxP3+ inducible Tregs producing TGF-β may down regulate T cell responses leading to the characteristic antigen specific anergy associated with lepromatous leprosy [17]. However, the role of Treg cells in leprosy in association with other subsets needs to be investigated.

Treg cells induced by the Programmed Death-1 (PD-1) pathway that assists in maintaining immune homeostasis and prevent autoimmune attack [18] may also lead to cellular anergy in lepromatous leprosy. PD-1 is a negative costimulatory molecule which exerts inhibitory effect on T cells by reducing cytokine production and cellular proliferation, with significant effects on IFN-γ, TNF-α and IL-2 production [18]. PD-1 may exert its influence on cell differentiation and survival directly through induction of apoptosis [19]. The PD-1-PD-L pathway also plays a key role in chronic infections as well as in the suppressive tumor microenvironment [20] by contributing directly to T-cell exhaustion and lack of immune response [21]. The importance of the PD-1-PD-L pathway in dampening the T cell responses during chronic infections has propelled the development of strategies to restore host immunity.

Treg cells are well known as inhibitor of various effector T cells such as IFN-γ producing Th1 and recently identified IL-17+ T helper 17 (Th17) cells [22]. Th17 is relatively a new lineage of effector T cells [23] and has been shown to be important in immune responses to various infectious and autoimmune diseases [24]. Th17 cells mediate their pro-inflammatory function by i) recruiting neutrophils, ii) activating macrophages, and iii) enhancing Th1 effector cells [25]. Much of the inflammatory damage previously ascribed to type 1 response is now thought to depend on IL-17 and IL-23 (the cytokine responsible for supporting Th17 response *in vivo*) [26]. A recent work on leprosy [27] identified CD4+ Th17 Cells in borderline cases and highlighted their importance in infectious diseases as well. However, little is known about the regulation of this effector T cell subset, especially in relation to Treg cells.

Treg and Th17 cells are therefore two lymphocyte subsets with counterbalancing effects on each other. Altered regulation between these two may contribute to the pathophysiology of infectious diseases by tipping the balance toward suppression of host immunity and subsequent dissemination of disease. Here, we attempted to identify and enumerate these cells in leprosy and the specific factors that impact their cell numbers and function and whether this can be modulated for benefit of the host. Recent findings [28] especially in autoimmune diseases, suggest that the equilibrium between Treg and Th17 cells might be pharmacologically restored for therapeutic benefit.

## Materials and Methods

### Patients and controls

A total of 40 newly diagnosed untreated leprosy patients (23 males, 17 females aged between 19–60 years) from Leprosy Clinic of the Department of Dermatology, All India Institute of Medical Sciences Hospital, New Delhi, India were included in the study (Table 1) and classified on the basis of Ridley-Jopling classification [1,2]. Study group included 20 borderline tuberculoid (BT), 20 lepromatous/borderline lepromatous (LL/BL) patients. Exclusion criteria included patients below 15 years of age, pregnant women, clinical evidence of anemia and other infections such as tuberculosis, HIV and helminthic infestation. PBMCs were investigated for flow cytometric analysis. 10 Healthy Contacts (HCs) were also included in the study.

**Table 1:** Clinical details of 40 newly diagnosed untreated leprosy patients and 10 healthy contact subjects. Patients were typed on the basis of Ridley Jopling classification [1], BI; Bacillary Index (mean of six lesional sites). M; male, F; female. BT: Borderline Tuberculoid, LL: Lepromatous Leprosy, HC: Healthy house hold contacts with long exposure to leprosy patients.

Clinical types	Number of Patients	Sex		Age	BI	Duration of leprosy
		M	F	(years)		(months)
Borderline Tuberculoid (BT)	20	12	8	20–57	0–0.5	1–36
Lepromatous/Borderline lepromatous Leprosy (BL/LL)	20	11	9	19–60	3.5–6	6–24
Healthy Contacts (HC)	10	6	4	22–40	-	-

### Ethics statement

Written informed consent was obtained from all study subjects. The research project was approved by the Institutional Ethics Committee (Ref.No.: IESC/T-339 /2010) at AIIMS, New Delhi.

### Antibodies and reagents

Monoclonal antibodies used for this study (anti-human CD4, CD25, CD45RO, CCR4, CCR5, CCR6, IL-10, IL-17, FoxP3 and purified antibodies against IL-10 and TGF-β) were obtained from BD Biosciences, CA, USA. Recombinant Soluble proteins of TGF-β, IL-6, IL-17, IL-22 and IL-23 were purchased from eBiosciences, San Diego, CA, USA. The whole cell lysate (WCL) of *M*. *leprae* was a kind gift from the Immunology laboratory of National Jalma Institute of Leprosy and Other Mycobacterial Diseases, ICMR, Agra, India.

### PBMC isolation and *in vitro* cultures

Peripheral blood mononuclear cells (PBMCs) were isolated from heparinized blood by Ficol hypaque (Sigma-Aldrich, St. Louis, USA) gradient centrifugation and suspended in RPMI-1640 (Caisson Laboratories, USA) supplemented with L-Glutamine (Sigma, USA), HEPES (Sigma, USA), antibiotics (Biological Industries, Israel) and 10% heat inactivated fetal calf serum (FCS) (Biological Industries, Israel) as per previously published protocol from our laborartory [9]. PBMCs were cultured (2x10^6^ cells/ml) with either PMA+Ionomycin (as positive control) for 6 hours or *M*. *leprae* antigen (WCL, 20μg/ml) for 48 hours at 5% CO_2_, 37°C and 1μM Monensin (Sigma, USA) added for last 6 hours. At the end of culture period, cells were stained for intracellular cytokines IL-10, IL-17, IFN-γ and the transcription factor FoxP3 and cell surface markers CD4, CD25, CD45RO, PD-1, PDL-1, CCR4 and CCR6. The cells were finally suspended in staining buffer and acquired on BD FACS Calibur; USA and analysis performed on Flowjo software.

### *In vitro* blocking experiments

*In vitro* blocking cell culture experiment was performed in leprosy patients with LL (n = 5) under different conditions, unstimulated vs. stimulated with leprosy antigen only vs. stimulated with antigen and blocked with purified antibodies against IL-10/TGF-β (BD Biosciences) alone or in combination. The experiment was performed in duplicates in 96 well culture plate at 5% CO_2_ and 37°C. After an incubation period of 72 hours, the readout was measured in terms of the cytokine IL-17 produced by CD4+ T cells through flow cytometric analysis.

In another experiment, PBMCs isolated from LL patients (n = 4) were incubated with or without blocking abs against PD-1 (5μg/ml, eBioscience), and/or PDL-1 (2μg/ml, eBioscience) to block the interaction between PD-1 and its ligand, in the presence or absence of *M*. *leprae* WCL and Brefeldin (GolgiPLUG 10μg/ml, Sigma, USA). Purified mouse IgG1 (final concentration of 10 mg/ml; eBioscience) was used as an isotype control. Cells were cultured in duplicates in 96 well culture plate at 5% CO_2_ and 37°C. After an incubation period of 72 hours, cells were examined for the percentage of IFN-γ and IL-17 secreting T cells by flow cytometry. Surface and intracellular staining protocol were performed as mentioned earlier. Stained cells were then acquired on BD FACS Calibur; USA and analysis performed in Flowjo software.

### Cell culture with recombinant proteins

For *ex vivo* cell culture assay using recombinant cytokines, freshly isolated PBMCs from LL patients (n = 5) were cultured for 72 hours in duplicates in 96 well culture plate at 5% CO_2_ and 37°C using *M*. *leprae* WCL antigen for stimulation. This was done with or without the presence of recombinant cytokines inducing Th17 population such as TGF-β, IL-6, IL-23 and the ones produced by Th17 like IL-17 and IL-22. Cytokines were obtained from eBiosciences and used at concentrations as recommended by the manufacturer in different wells. At the end of culture period, flow cytometry was used to measure the frequency of FoxP3+ CD4+ cells on one hand with simultaneous IL-17 production by CD4+T cells.

### Statistical analysis

FACS analysis was performed with Flowjo software (Tree Star, Oregon, USA). Statistical analysis was performed using GraphPad Prism5 software (La Jolla, CA, USA) applying Student’s t test for unpaired samples. Values of p<0.05 were considered significant.

## Results

### Enhanced IL10 production and CCR4 expression by Treg cells in BL/LL patients

**Fig 1:**
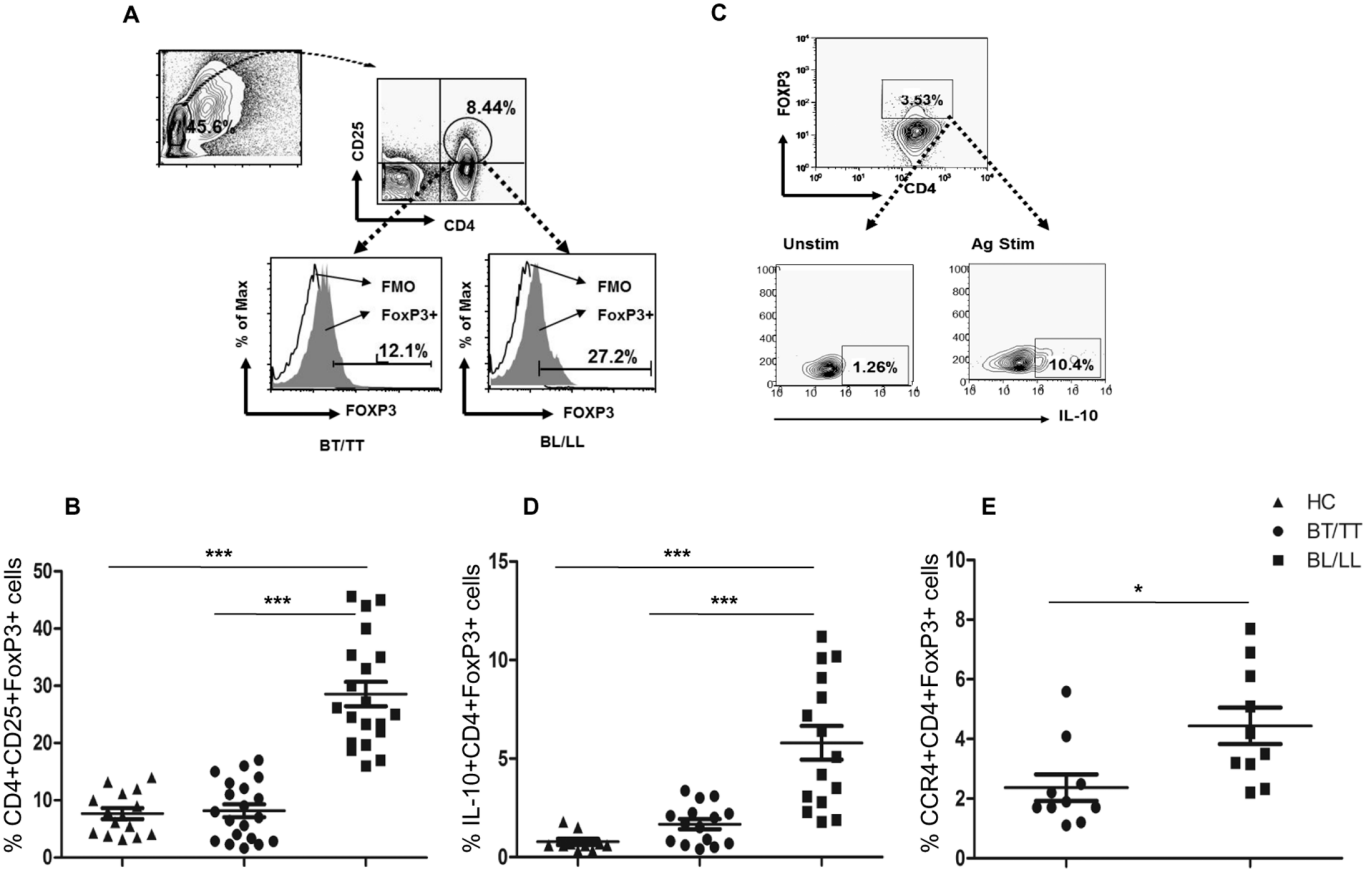
T regulatory cells in Leprosy. A) Representative FACS plot showing enumeration of Treg cell frequency (CD4+, CD25+ and FoxP3+) in BT/TT vs. BL/LL patient B) Total frequency of circulating Treg (CD4+, CD25+ and FoxP3+) cells in PBMCs isolated from peripheral blood of BT/TT vs. BL/LL patients (n = 20) vs. Healthy Contacts (n = 10). C) Representative flow cytometric analysis showing IL-10 production following *in vitro* stimulation with *M*. *leprae* (WCL) vs. unstimulated for 48 h in a Leprosy patient with LL. D) Scatter Plots are showing total IL-10 production in gated CD4+ FoxP3+ cells in BT/TT vs. BL/LL (n = 15) vs. Healthy Contacts (n = 10). E) CCR4 surface expression on gated CD4+ FoxP3+ cells in different groups of Leprosy patients (BT/TT vs. BL/LL), (n = 10). Each dot represents a single individual. Median values are shown in each set while P value< 0.05 was considered to be significant. Total number of cells analyzed by flow cytometry were 500,000. Data analysis was performed with flowjo software. Statistical analysis was done using Student’s t test for unpaired samples.

In order to identify the Treg cells in human leprosy, immunophenotyping was done for the markers CD4, CD25 and FoxP3 (Fig 1A) on PBMCs derived from leprosy patients (BT/TT vs. BL/LL) (n = 15 in each group) and healthy contacts (HCs, n = 10). Peripheral representation of Treg cells was profoundly increased among BL/LL as compared to BT/TT patients and HCs (p = 0.0002, p<0.0001 respectively) (Fig 1B). Enrichment of Treg cells among BL/LL patients hints towards their important role in the characteristic immune suppression observed in them. To evaluate the function of enriched Treg cells in BL/LL patients, we measured their intracellular IL-10 production by flow cytometry (Fig 1C), using the whole cell lysate of *M*. *leprae* (WCL) for antigen specific stimulation. Percentage of IL-10+ Treg cells (CD4+FoxP3+) was significantly higher among BL/LL patients (Fig 1D) compared to that of BT/TT patients (P = 0.0003). The increased frequency of IL-10 producing Treg cells in BL/LL patients indicates their suppressive role in a contact-independent manner via release of IL-10. The surface expression of the chemokine receptor, CCR4 was also significantly high on Treg cells in leprosy patients with BL/LL (Fig 1E). This again highlights that Treg cells are possibly recruited by means of this receptor to the lesional sites in BL/LL patients, wherein they mediate their characteristic suppressive action.

### Elevated IL-17 and CCR6 expression on CD4+CD45RO+ cells in BT/TT patients

**Fig 2:**
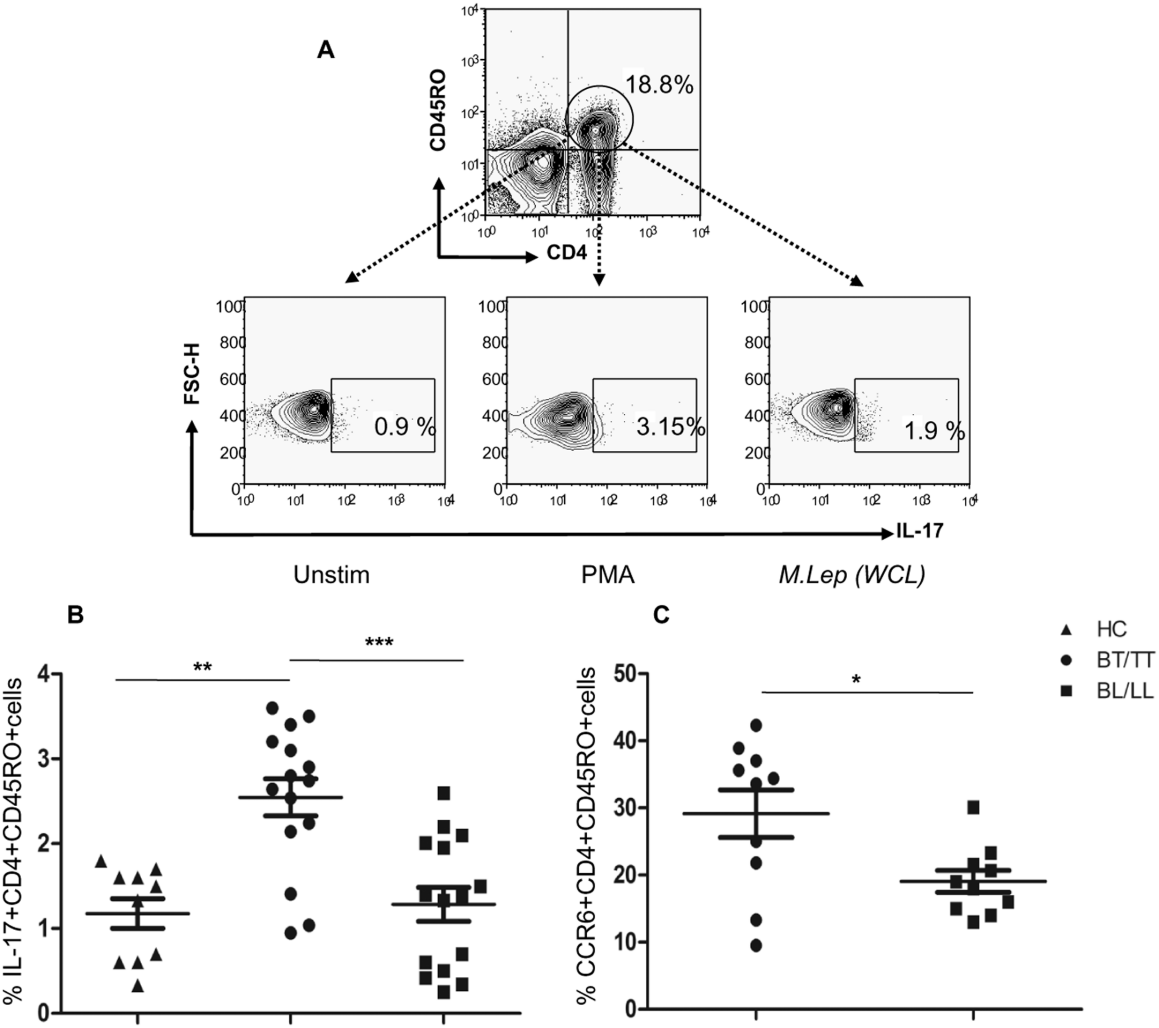
Identification of Th17 cells in Leprosy. A) Representative flow cytometric analysis showing IL-17 production on gated CD4+ CD45RO+ memory T cells following *in vitro* stimulation with *M*. *leprae* (WCL) vs. unstimulated for 48 h in a Leprosy patient with BT. 6 h PMA stimulation was used as positive control. B) Scatter plots are showing cumulative IL-17 production in gated CD4+ CD45RO+ cells in different groups of Leprosy patients (BT/TT vs. BL/LL) and Healthy Contacts (n = 10). Each dot represents a single individual of BT/TT (n = 15) and BL/LL (n = 15) patient. C) Scatter graphs are showing CCR6 surface expression on gated CD4+CD45RO+ cells in different groups of Leprosy patients (BT/TT vs. BL/LL) (n = 10). Median values are shown in each set while P value< 0.05 was considered to be significant. Total number of cells analyzed by flow cytometry were 500,000. Data analysis was performed with flowjo software. Statistical analysis was done using Student’s t test for unpaired samples.

The Th17 cells are distinct from the classical Th1 and Th2 cell subsets and identified by production of signature cytokine, IL-17. PBMCs isolated from leprosy patients were stimulated with PMA+Ionomycin (as positive control) and *M*. *leprae* antigen (WCL) separately. Intracellular staining for IL-17 (Fig 2A) on CD4+CD45RO+ T cells showed significantly higher frequency of Th17 cells in BT/TT (P = 0.0005) as compared to BL/LL patients (Fig 2B). This hints towards a possible role of these cells in up-regulation of cell mediated immunity in BT/TT patients leading to containment of disease in the infected host. We also evaluated the expression of CCR6, a well-known chemokine receptor on Th17 cells and observed significant levels of CCR6 surface expression on gated CD4+CD45RO+ cells (P = 0.03) in BT/TT as against BL/LL patients (Fig 2C). This indicates that Th17 cells plausibly get recruited at the pathologic sites of BT/TT patients and potentiates the Th1 like effector T cells.

### IL-10+ Treg cells inversely correlate with Th17 cells in BL/LL patients

**Fig 3:**
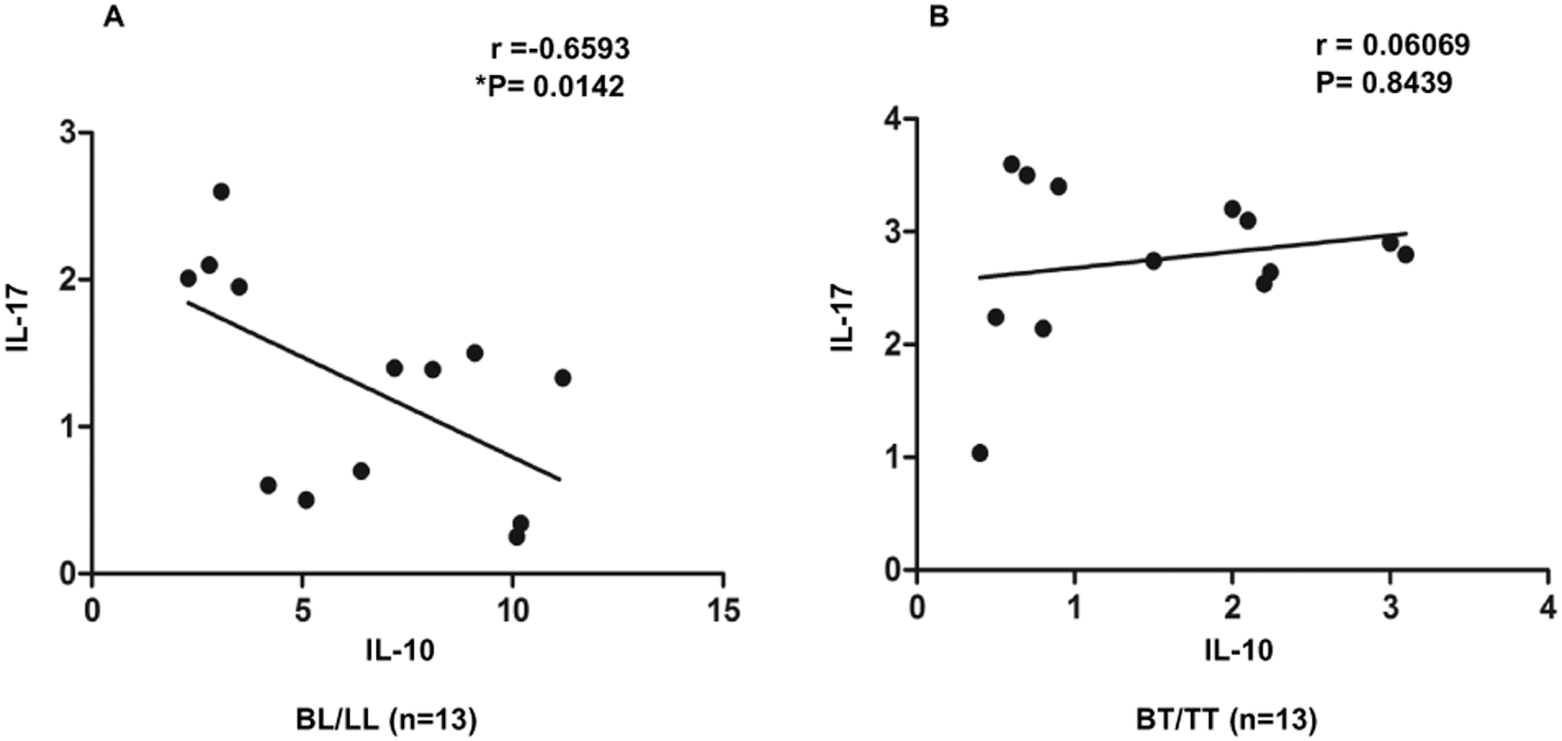
Inverse Correlation between IL-10 and IL-17 in BL/LL. A) Treg derived high IL-10 negatively correlates with low IL-17+ CD4 T cells in BL/LL (n = 13) signifying polarized immunity in leprosy. B) No correlation was found in BT/TT (n = 13). Correlation was done using Spearman rank correlation coefficient; r value was equal to -0.65 while p value was significant at 0.01 in case of BL/LL.

Both Treg and Th17 cells are known to have important implications in determining the host immunity and subsequent disease manifestation. Hence, we attempted to correlate these two cell types, in both BT/TT and BL/LL patients. In case of BL/LL, there existed a significant inverse correlation (r = -0.68, P = 0.03) between these two T cell subsets (Fig 3A), which indicates that high IL-10 production in BL/LL correlates with suppressed state of inflammatory immune response as evidenced by lesser number of IL-17+ Th17 cells. However, in BT/TT patients no correlation was found between these two cytokine producing T cell subsets (Fig 3B). Our results indicate that these two cell types may play critical roles in shaping the host immune response and relative frequencies of IL-10 vs. IL-17 producing T cells can determine the disease outcome. This could also be exploited as a sensitive and specific pathologic biomarker for disease susceptibility in human hosts.

### IL-10 & TGF-β mediated suppression of Th17 cells in BL/LL patients

**Fig 4:**
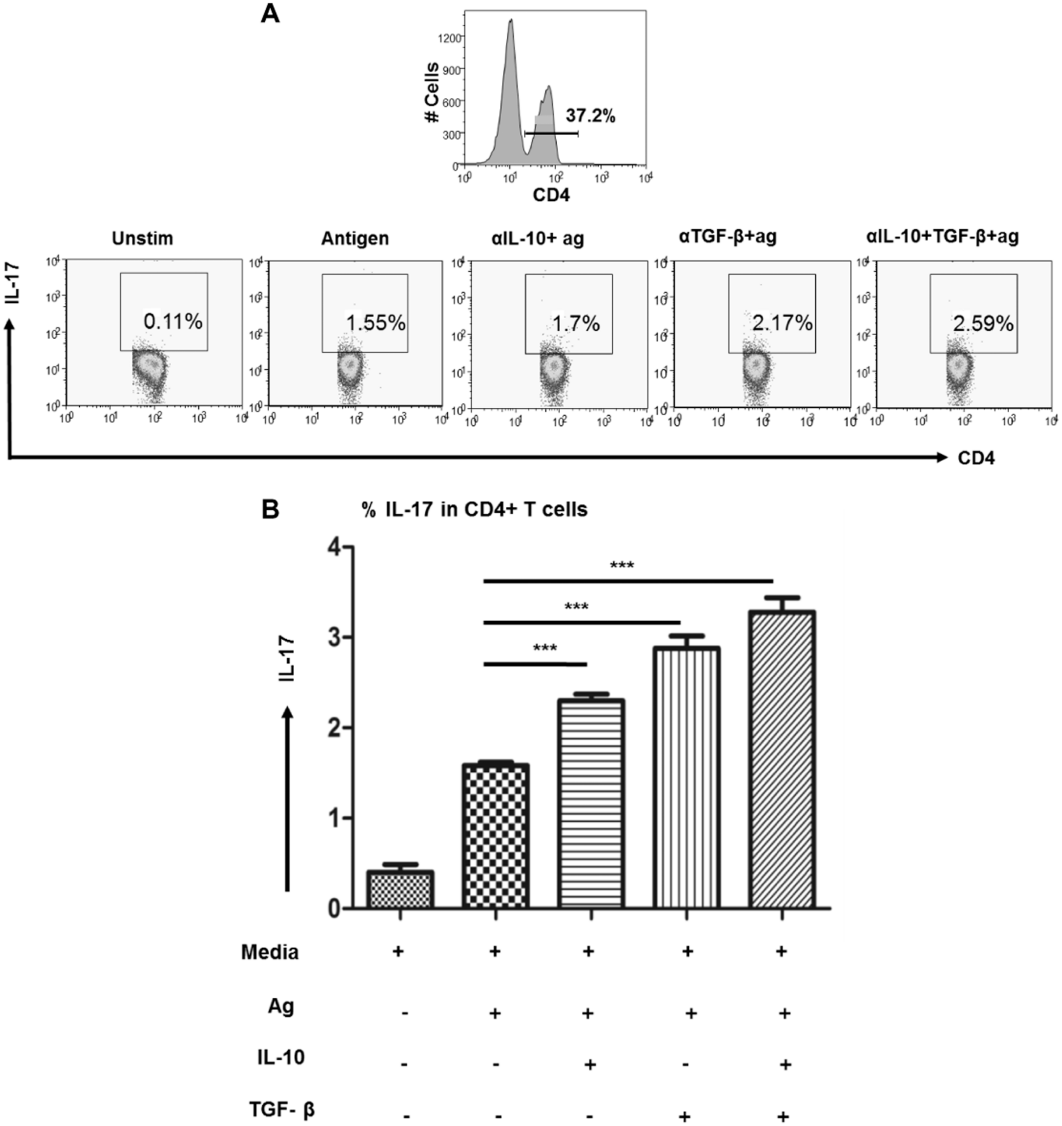
IL-10/TGF-β suppresses Th17 in BL/LL. A) Flow cytometric analysis showing the increased production of IL-17 on total gated CD4+ T lymphocytes in a leprosy patient with LL under different conditions, Unstimulated vs. Stimulated with leprosy antigen only vs. Stimulated with antigen and blocked with antibodies against IL-10/TGF-β alone or in combination. B) Cumulative data of BL/LL (n = 5) patients are shown in the bar graphs with the same conditions as above. Readout was taken in terms of IL-17 by CD4+ T cells at the end of 72 h. Mean with SEM are shown in each bar while P value< 0.05 was considered to be significant. Total number of cells analyzed by flow cytometry were 500,000. Data analysis was performed with flowjo software. Statistical analysis was done using Student’s t test for unpaired samples.

Significant association of the suppressive cytokine IL-10 produced by CD4+FoxP3+ cells with IL-17 by CD4+CD45RO+ T cells in BL/LL patients prompted us to investigate the effect of blocking the cytokines produced by Treg cells, mainly IL-10 and TGF-β on Th17 cells. In BL/LL patients (n = 5), *in vitro* blockade of IL-10 and TGF-β alone resulted in elevated frequency of Th17 cells, while dual blockade led to significant increase in IL-17+ T cells (Fig 4A and 4B). This suggests restoration of the effector immune response in terms of IL-17 production by manipulating the Treg cell derived suppressive cytokines, IL-10 and/or TGF-β in BL/LL patients.

### Th17 cytokines reduces the frequency of FoxP3+ Treg cells in BL/LL patients

**Fig 5:**
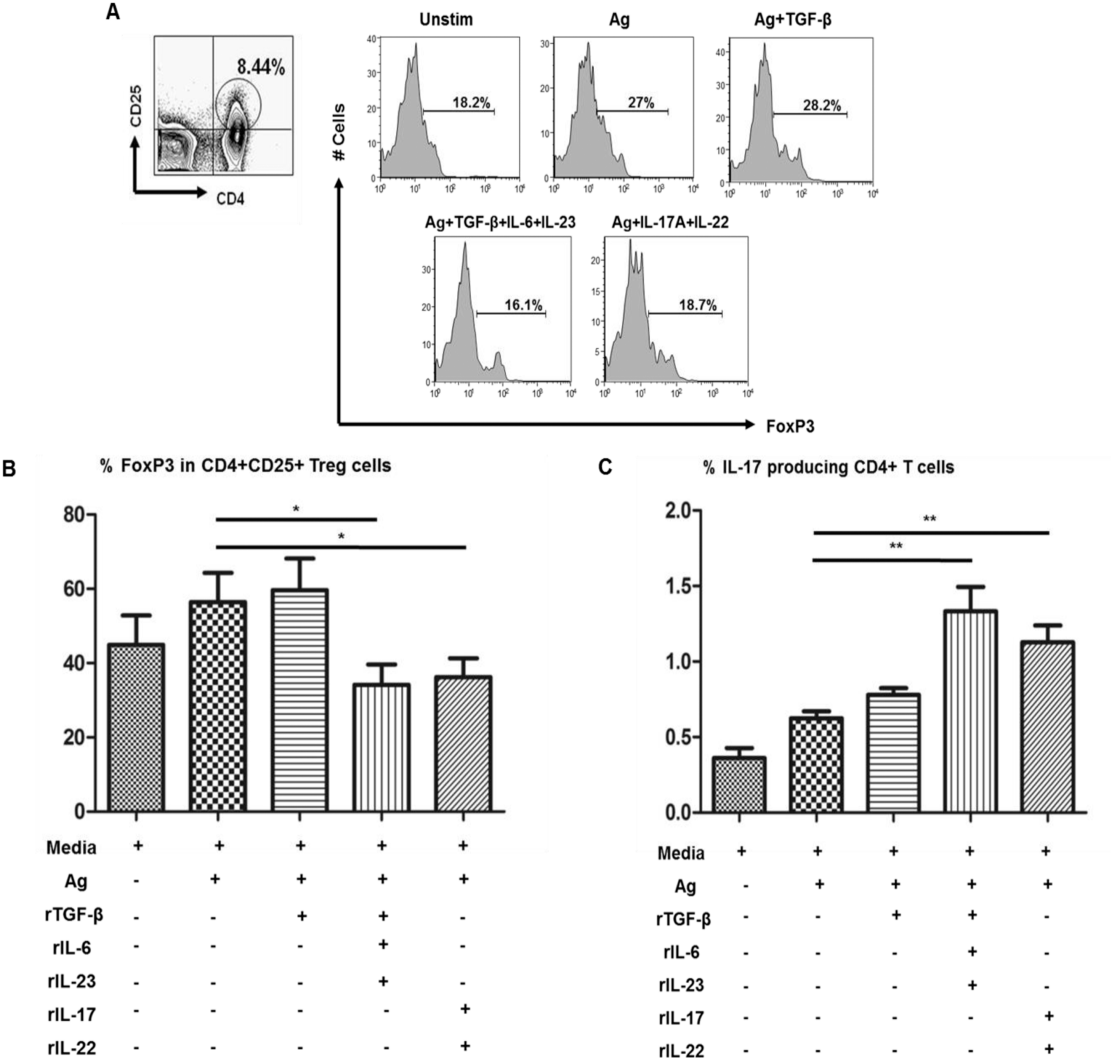
Effect of Th17 cytokines on FoxP3+Treg cells in BL/LL. A) PBMCs were cultured for 72 h in different conditions with or without the presence of cytokines inducing Th17 population such as TGF-β, IL-6, IL-23 and those secreted by Th17, IL-17 and IL-22 in different wells and readout was measured in terms of FoxP3+ on CD4+CD25+ cells on one hand and simultaneous IL-17 production by CD4+T cells. B) Bar graphs show the cumulative data in BL/LL (n = 5) of FoxP3+ expression on CD4+ CD25+ cells as against C) IL-17 production by CD4+T cells under the same conditions as mentioned above. Mean with SEM are shown in each bar while P value< 0.05 was considered to be significant. Total number of cells analyzed by flow cytometry were 500,000. Data analysis was performed with flowjo software. Statistical analysis was done using Student’s t test for unpaired samples.

Differentiation of naive T cells towards a Th17 phenotype is induced by several cytokines including TGF-β, IL-1β, IL-6, IL-21, and IL-23 in mice and humans. Hence, we performed *ex vivo* cell culture assays using PBMCs from BL/LL patients (n = 5) in the presence or absence of Th17 inducing cytokines, TGF-β, IL-6, IL-23 and cytokines produced by Th17, IL-17 and IL-22 (Fig 5A). Results showed marked reduction in the frequency of FoxP3+ cells (Fig 5B) in the presence of Th17 inducing cytokines (TGF-β, IL-6, IL-23) and also in presence of cytokines produced by Th17 (IL-17 and IL-22). Simultaneously, there was an increase in IL-17 producing CD4+ cells in BL/LL patients in these same experimental conditions (Fig 5C), hinting that Treg cells are affected by the cytokine milieu they are exposed to. Our result suggests that by means of these cytokines associated with Th17 cells (TGF-β, IL-6, IL-17, IL-22 and IL-23), it is possible to alter the number of Treg cells in BL/LL patients.

### Higher PD-1 on Treg cells and PDL-1 on APCs in BL/LL patients

**Fig 6:**
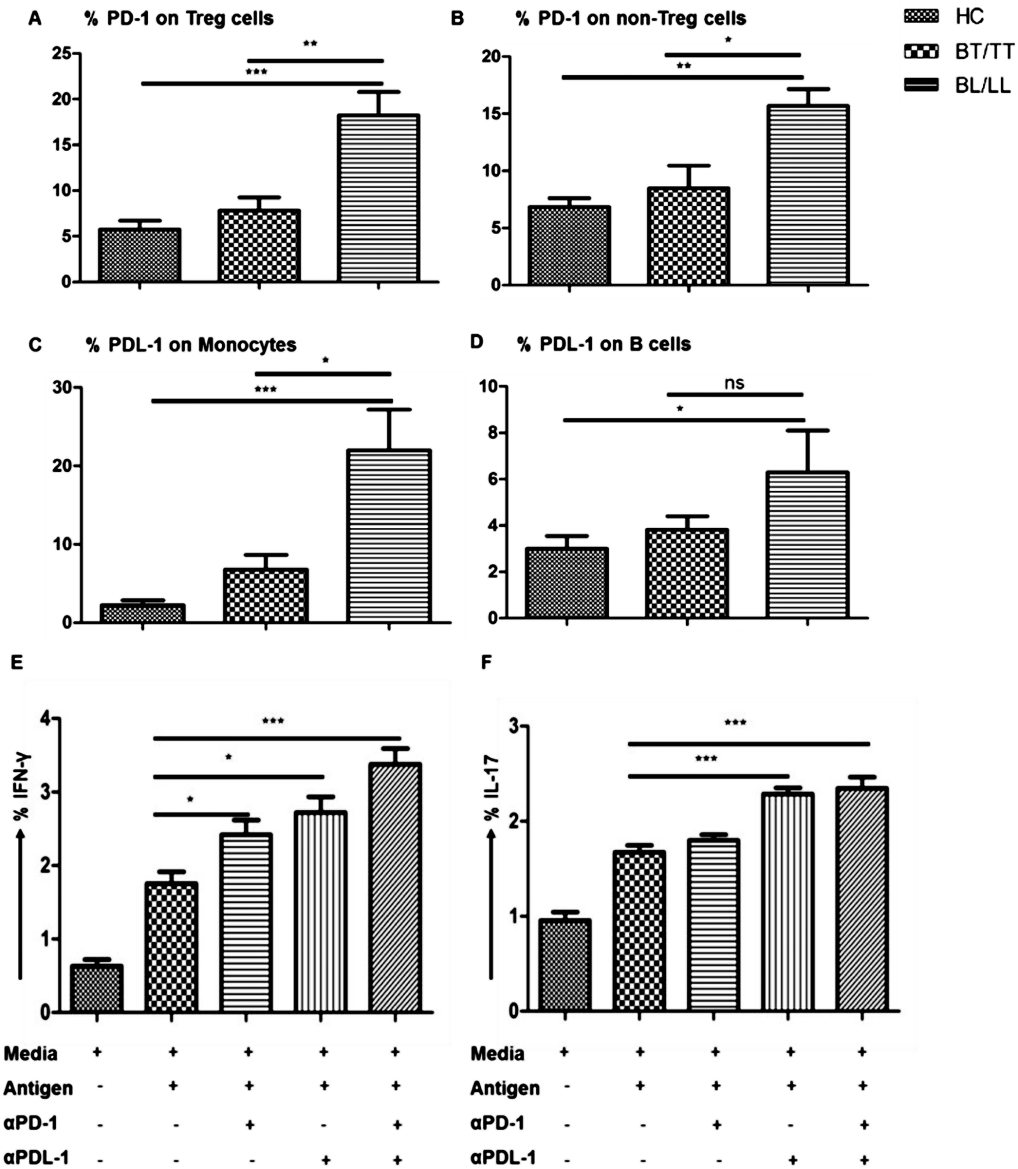
PD-1/PDL-1 Interaction Suppress IFN-γ and IL-17 Effector cytokines in BL/LL. A) Percentage frequency of PD-1 expression on CD4+FoxP3+ Treg cells vs. B) non Treg cells, (CD4+FoxP3-) in BT/TT vs. BL/LL (n = 10) patients and healthy contacts (n = 10). Percentage frequency of PDL-1 expression on APCs, C) on CD14+ Monocytes D) on CD19+ B cells in BT/TT vs. BL/LL (n = 10) patients and healthy contacts (n = 10) in PBMCs isolated from peripheral blood. Each bar represents a single category. Error bars are shown in each set while P value< 0.05 was considered to be significant. Bar graph representation showing the increased production of E) IFN-γ and F) IL-17 on total gated CD4+ T lymphocytes in BL/LL patients of leprosy (n = 4). PBMCs isolated from peripheral blood were blocked with α-PD-1 or with α-PDL-1 monoclonal blocking antibodies separately or in combination and kept in culture for 72 hours with or without *M*. *leprae* WCL antigen. From cultured cells, T cells were analyzed for effector cytokine production by flow cytometry. Total number of cells analyzed by flow cytometry were 500,000. Each bar represents a single category. Mean with SEM are shown in each bar while P value< 0.05 was considered to be significant. Data analysis was performed with flowjo software. Statistical analysis was done using Student’s t test for unpaired samples.

Moreover, we looked for surface expression of the known suppressive molecule, PD-1 on Treg and non Treg cells by flow cytomtery. Significantly higher percentage of Treg cells (as well as non Treg cells) of BL/LL patients expressed PD-1 on their surface compared to that of BT/TT patients and healthy contacts (Fig 6A and 6B). However, percent PD1+ cells were more in Treg cells relative to FoxP3- T cells (non-Treg) in BL/LL cases. Subsequent assessment of its ligand (PDL-1) revealed higher frequency of CD14+ Monocytes and CD19+ B cells expressing the PDL-1 (Fig 6C and 6D). This data indicates towards a possible role of PD-1/PDL-1 interaction in Treg cell mediated suppression of effector T cells function in BL/LL patients.

To validate the role of PD-1 pathway, we performed *in vitro* experiments using PBMCs from BL/LL patients (n = 4) stimulated with *M*. *leprae* antigen in presence or absence of blocking monoclonal antibodies against PD-1/PDL-1 and measured the intracellular inflammatory cytokines IFN-γ and IL-17 production by CD4+T cells. When PD-1 pathway was blocked, significantly higher number of T cells produced both the pro-inflammatory cytokines, IFN-γ and IL-17 indicating that PD-1/PDL-1 interaction inhibits Th1 and Th17 effector cells in BL/LL patients (Fig 6E and 6F). Considering the higher frequency of PD1+ Treg cells in the BL/LL cases, we envisage that PD1 expressed by the Treg cells dampens the T cell function in these patients. However, PD1 expressed on other subsets of cells may also contribute to the suppression in these cases.

## Discussion

Regulatory T (Treg) cells are essential for maintaining peripheral tolerance, preventing autoimmune diseases and limiting chronic inflammatory diseases [29, 30]. However, in case of chronic infections, they also dampen the host immune response against the pathogen(s) [31]. During an infection, immune regulation is the result of the host’s immune response to the infection in a bid to maintain or restore a homeostatic environment [32]. This property is exploited by the pathogen for evading host effector response hence promoting its survival [33]. During *M*. *leprae* infection varied effector T cell response regulates such balance in a way either containing the pathogen or rendering their survival via immune suppression as in BT/TT and BL/LL respectively [34]. Treg cells suppress effector T cells like Th1 and Th17 cells both by contact dependent as well as contact independent manner. Inhibitory cytokines, IL‑10 and TGF-β, have been the focus of considerable attention as mediators of Treg cell induced suppression [15, 16].

Differential trafficking of these regulatory T cells to the diseased sites are thought to be under the influence of tissue chemokine response [35] elicited at the site of LL lesions. Recently we demonstrated that tissue chemokine response at the lepromin DTH site and lesions of various forms of leprosy determines the recruitment of effector T cells at the lesional levels in leprosy patients [36]. Some of these subsets have been demonstrated to be hierarchic in nature and exert significant influence on the effector T cells thus regulating the immune response at the pathologic site(s). These include the FoxP3 positive regulatory T cells and our findings validate their critical role in dampening the host effector T cell response during intracellular infections such as tuberculosis [9] and leishmaniasis [11]. A study in leprosy revealed that FoxP3+ Treg cells that produce TGF-β down regulate the T cell responses and results in the characteristic antigen specific anergy observed in LL cases [17]. However, the role of PD1 in Treg mediated immunosuppression in BL/LL still remains to be studied.

In our study, the frequency of Treg cells showed >5-fold increase in case of BL/LL compared to BT/TT and HCs (p = 0.0002, p<0.0001 respectively). This is in corroboration with previous data from leprosy [37, 38]. These Treg cells also produce significant amounts of IL-10 in BL/LL vs. BT/TT indicating that these cells are suppressive in nature. Further analysis of surface expression of the chemokine receptor, revealed higher frequency of CCR4+ Treg cells in BL/LL cases as compared to BT/TT. Considering the role of CCR4 in Treg cell homing, we propose that it possibly aids in rapid recruitment of Treg cells at BL/LL lesions. Such local enrichment of Treg cells may facilitate dissemination of disease.

For long, Th1 cells were considered to be the major effectors in multiple autoimmune diseases, while Th2 cells were involved in atopy and asthma [39,40,41]. Recently Th17 cells have been implicated in inflammatory diseases [42]. These cells produce IL-17A (also referred to as IL-17), IL-17F, and IL-22, cytokines involved in neutrophilia, tissue remodeling and repair, and production of antimicrobial proteins [24,25]. In our study on leprosy, significantly higher frequency of IL-17+ was found on CD4+CD45RO+ cells in BT/TT (P = 0.0005) as opposed to BL/LL patients. This is indicative of their possible role in inflammatory T cell response observed in BT/TT patients. These cells are also known to express the chemokine receptor CCR6 and we found its expression of Th17 cells (P = 0.03) in BT/TT as against BL/LL. This indicates possible homing of IL-17+ effector T cells in the BT/TT lesions through CCR6. Thus, chemokine receptor mediated reciprocal recruitment of Treg vs. Th17 cells may determine the lesional level immune response and clinical manifestation (localized vs. disseminated in BT/TT vs. BL/LL respectively).

Imbalance between Th17 and Treg cell function may be critical in the immunopathogenesis of many disease states [43]. We therefore, correlated the IL-10+ Treg cells with IL-17+ helper T cells and found a significant inverse correlation in BL/LL patients. This indicates that tilting of the balance towards Treg cells may be responsible for the cellular anergy observed in these patients. However, such correlation among BT/TT patients was not significant. This encouraged us to block the Treg cell secreted cytokines, IL-10/TGF-β *in vitro* in the PBMC culture of BL/LL patients and see their impact on the effector immune response in terms of IL-17. Blocking of each of these cytokines alone or in combination resulted in rescue IL-17+ T cells in BL/LL. This indicates that by negating the influence of suppressive cytokines we can successfully restore the beneficial effector T cells response in lepromatous leprosy cases.

We further evaluated the impact of Th17 associated cytokines, both Th17 inducing (TGF-β, IL-6, IL-23) and those secreted by Th17 cells (IL-17 and IL-22) on Treg cells. Presence of inducing (TGF-β, IL-6, IL-23) and secreted (IL-17 and IL-22) cytokines markedly reduced the number of Treg cells determined by reduced FoxP3 positivity on CD4+CD25+ cells with simultaneous increase of IL-17+ CD4 T cells. This reduction was however, not seen in cells stimulated with TGF-β alone. Lesser frequency of FoxP3+ Treg cells may also be due to possible apoptosis of these cells in a relatively hostile microenvironment. This indicates that antigen-specific Treg cells are largely dependent on the cytokine milieu they are exposed to. Tipping the balance of Treg cells towards Th17 cell phenotype via alteration of cytokine milieu can result in restoration of *M*. *leprae* specific effector T cell response and may therefore constitute possible therapeutic strategy for reversal of immune unresponsiveness in lepromatous leprosy.

T cell responses during parasitic infections are tightly controlled by co-stimulatory or co-inhibitory molecules [44,45,46]. PD-1-PDL-1 interaction inhibits the effector T cell functions such as, proliferation, cytokine production and survival, thus balancing the tolerance, autoimmunity, infection, and immunopathology [47,48]. A recent study showed that *in vitro* blockade of PD-1 signaling with the specific antibody enhanced IFN-γ production by T cells of TB patients on stimulation with *M*. *tuberculosis* antigen [49]. These findings raise the possibility that the antigen-specific T-cell response is impaired by some inhibitory mechanism, thereby allowing mycobacterial persistence which was recently demonstrated in our laboratory [50]. When we analyzed for the surface expression of PD-1 on Treg and non Treg cells, we found that PD-1 is significantly elevated in BL/LL as compared to BT/TT patients. Even the ligand PDL-1 was higher on CD14+ monocytes and CD19+ B cells suggesting their role in host immune impairment in leprosy. This may be one of the contact-dependent mechanisms utilized by Treg cells for immune suppression. The efficacy of this pathway was firmly established after blockade of PD1-PDL1 interaction increased the *M*. *leprae* specific IL-17 and IFN-γ producing T cells in BL/LL patients. Hence, inhibition of this pathway may be an efficient means to revive the host immune response.

Taken together, our study highlights the importance of the equilibrium between Treg and Th17 cells in determining host immunity. Providing a balanced level of function for these two important cell subsets is the key to achieving an appropriate level of parasite control without inducing immunopathology. This would be a major goal in the management of this still-challenging infectious disease.
